# Predicting Car-Engine Manufacturing Quality with Multi-Sensor Data of Manufacturing Assembly Process

**DOI:** 10.3390/s26051651

**Published:** 2026-03-05

**Authors:** Xinyu Yang, Qianxi Zhang, Junjie Bao, Xue Wang, Nengchao Wu, Qing Tao, Haijia Wu, Li Liu

**Affiliations:** 1 School of Mechanical Engineering, Xinjiang University, Urumqi 830017, China; yangxinyu@stu.xju.edu.cn (X.Y.); 107552404373@stu.xju.edu.cn (Q.Z.); 107552404289@stu.xju.edu.cn (J.B.); 107552404312@stu.xju.edu.cn (X.W.); wnc@stu.xju.edu.cn (N.W.); taoqing@xju.edu.cn (Q.T.); 2 Chongqing Municipal Economy and Information Technology Commission, Chongqing 401121, China; wuhaijia@jjxxw.cq.gov.cn; 3 School of Big Data & Software Engineering, Chongqing University, Chongqing 400030, China

**Keywords:** heterogeneous multi-sensor data, sensor data fusion, feature extraction, industrial IoT, engine manufacturing quality prediction

## Abstract

Car engine quality control is fundamentally hindered by extremely high-dimensional, noisy, and imbalanced multi-sensor data. To overcome these challenges, this paper proposes an edge-deployable diagnostic and predictive framework. First, a Sparse Autoencoder (SAE) maps over 12,000 distributed manufacturing parameters into a robust latent space to filter instrumentation noise. Second, for defect classification, a Class-Specific Weighted Ensemble (CSWE) tackles extreme class imbalance by aggressively penalizing majority-class bias, improving defect interception recall by 7.72%. Third, for transient performance tracking, an Adaptive Regime-Switching Regression (ARSR) replaces manual phase selection with unsupervised regime routing to dynamically weight local experts, reducing relative prediction error by 12%. Rigorously validated across three diverse public datasets (NASA C-MAPSS, AI4I, SECOM) and a physical H4 engine assembly line, the framework achieves an ultra-low inference latency of 80±3 ms, practically reducing the engine rework rate by 7.2%.

## 1. Introduction

Automobiles are a cornerstone of modern discrete manufacturing [1,2]. As the core power unit of a vehicle, the internal combustion engine’s assembly quality directly dictates the holistic performance, safety, and manufacturing cost of the final product [3]. In contemporary production environments, predicting and verifying engine manufacturing quality at the End-of-Line (EOL) testing stage is critical. Early identification of mechanical or thermodynamic anomalies effectively minimizes rework overhead, optimizes production efficiency, and prevents defective units from escaping into the market [4,5].

However, engine assembly lines face profound challenges due to the extreme heterogeneity of multi-sensor data. Modern test benches generate massive, high-frequency data streams encompassing disparate physical domains, such as dynamic torque, rotational speed, oil pressure, and pneumatic leakage rates. These heterogeneous sensors exhibit massive scale discrepancies (e.g., kPa vs. Nm) and varying degrees of non-stationary electromagnetic interference. Traditional quality control protocols, which predominantly rely on rigid empirical thresholds or manual expert inspection, are fundamentally inadequate for deciphering the high-dimensional, non-linear correlations embedded within such complex data. Consequently, developing intelligent, data-driven diagnostic models has become a primary objective for advancing smart manufacturing.

Leveraging this heterogeneous data presents two primary algorithmic bottlenecks. First, the raw sensor arrays are characterized by high dimensionality and severe multicollinearity (redundant features) [6,7,8], demanding robust feature extraction methodologies that can filter noise without discarding critical transient physical indicators. Second, effective data fusion is heavily impeded by the non-stationary nature of engine testing. Engines undergo deterministic, discrete phase transitions (e.g., from Start-up, to High-speed, to Idle) [9]. Existing global approximation models typically struggle to adapt to these abrupt statistical shifts, and their reliance on complex deep learning architectures often exceeds the computational and latency constraints of industrial line-side edge servers.

To bridge these gaps, this study proposes a unified, lightweight, and highly adaptive diagnostic framework tailored for complex engine assembly lines. We integrate Adaptive Standardization with a Sparse Autoencoder (SAE) optimized via the L-BFGS algorithm. This combination effectively unifies physical scales, suppresses background noise, and extracts compact latent features while maintaining strict edge-computing viability. Furthermore, to address the distinct requirements of defect classification and continuous performance tracking, we propose two specialized diagnostic strategies designed to handle extreme class imbalance and non-stationary operating regimes.

The primary contributions of this study are summarized as follows:

1. Unified High-Dimensional Feature Extraction: We developed a robust pipeline utilizing a Sparse Autoencoder (SAE) to project extreme high-dimensional ($N_{total}>$ 12,000), heterogeneous sensor data into a scale-unified latent space. This module effectively filters out industrial electromagnetic interference while preserving critical mechanical dynamics, without relying on complex, latency-heavy deep network architectures.
2. Class-Specific Weighted Ensemble (CSWE) for Asymmetric Defect Detection: To address the severe class imbalance inherent in modern automotive quality control (where defect rates are intrinsically extremely low), we introduce the CSWE strategy. Unlike standard ensemble methods, CSWE utilizes a mathematically rigorous, recall-oriented objective function designed to aggressively penalize majority-class bias, thereby significantly maximizing the recall rate for identifying rare defective engines without compromising overall stability.
3. Adaptive Regime-Switching Regression (ARSR) via Unsupervised Routing: To overcome the underfitting limitations of global monolithic models under non-stationary dynamics, we designed the ARSR framework. For latent or unlabelled environments, ARSR employs an unsupervised regime routing mechanism (via K-Means clustering on SAE latent features) to automatically discover hidden operational phases and dynamically weight the optimal local experts, providing a principled adaptive architecture.
4. Physics-Informed Edge-Optimized Deployment: The proposed framework was successfully deployed as a real-time system on a physical automotive assembly line. For this specific physical application, the regime routing utilizes deterministic, physics-based hard-switching (RPM boundaries) as a deliberate architectural simplification. This strict edge-optimization ensures an ultra-low inference latency of under 80 ms (satisfying Takt time constraints) and has practically demonstrated its engineering viability by reducing the engine rework rate by 7.2%.

The remainder of this paper is organized as follows to ensure clarity and logical progression: Section 2 reviews related work in data preprocessing and multi-sensor fault detection. Section 3 details the proposed methodology, including the SAE, CSWE, and ARSR modules. Section 4 describes the industrial data acquisition and experimental setup. Section 5 presents the objective experimental results on both public benchmarks and the industrial dataset. Section 6 provides an in-depth discussion of the framework’s mechanisms, engineering boundaries, and physical interpretations. Finally, Section 7 concludes the paper.

## 2. Related Work

### 2.1. Data Preprocessing

Preprocessing heterogeneous multi-sensor data is essential for engine assembly line fault diagnosis, as it directly impacts data integrity and feature interoperability [10]. Despite extensive research, industrial environments continue to grapple with high dimensionality, sparse labeling, and significant feature redundancy [6].

To manage high-dimensional complexity, traditional statistical methods and emerging deep learning techniques offer different trade-offs. For instance, PCA-based variants like PCA-DP and CMPCA-DP have been utilized to balance privacy with data utility through effective aggregation and noise reduction [11]. Similarly, graph-representation-based feature selection has shown promise in improving efficiency and mitigating overfitting [12]. However, a persistent bottleneck remains: standard linear transformations often fail to resolve the complex non-linear couplings in sensor streams, while graph-based architectures frequently demand computational resources that exceed the limitations of real-time industrial deployment [13,14].

In the realm of noise suppression, specialized algorithms such as enhanced genetic algorithms, hybrid SEMD–ISSA–KELMC frameworks [15], and Krylov subspace techniques [16] have successfully addressed interference in electromagnetic or vibration datasets. Yet, these signal-specific solutions are often ill-equipped for the extreme scale inconsistencies—such as the vast dimensional gaps between torque (Nm) and fluid pressure (kPa)—intrinsic to engine assembly data. Furthermore, they rarely explore the deep, cross-modal correlations necessary for holistic manufacturing quality prediction.

Recent advances in anomaly detection, such as the MAGMM algorithm [17] and the BBTGO meta-heuristic [13], attempt to capture non-linear features while preserving critical spatial information. Nevertheless, the high computational complexity of these frameworks hinders their execution on resource-constrained edge servers, where sub-100ms latency is a hard requirement [13]. This operational gap necessitates a more lightweight yet robust solution, such as the SAE framework optimized via L-BFGS proposed in this study.

The scarcity of labeled samples presents another critical hurdle [18]. While optimization paradigms involving DWT, SSA, or adaptive three-stage strategies have improved signal quality [19,20,21], many remain tethered to rigid prior assumptions about data distribution. In actual industrial settings, these methods often fail to adapt to extremely sparse annotations or leverage the massive, continuous streams of unlabeled sensor data.

Current fusion techniques, ranging from wavelet-PCA combinations [22] to improved MPGA-ACO-BP neural networks [6], have made strides in integrating multi-source data. However, these systems often struggle with physical domain alignment (e.g., mechanical vs. thermodynamic) and lack the dynamic weight allocation required to handle abrupt signal fluctuations across the engine’s discrete operating phases [10]. This study addresses these gaps by introducing an unsupervised feature learning approach that capitalizes on unlabeled data while maintaining high-precision, phase-adaptive modeling.

### 2.2. Multi-Source Sensor Data Fault Detection Algorithms

Although existing multi-sensor fault diagnosis methods have advanced significantly, they exhibit critical limitations in heterogeneous feature fusion and dynamic adaptability. Deep learning architectures, such as ResNet18 [23], CNN-LSTM [24], Transformer-based frameworks [25,26], and Digital Twin models [27], have facilitated cross-source data interaction. However, they share two primary drawbacks: First, they often struggle with physical scale disparities (e.g., mechanical torque versus sealing leakage) during multi-modal data fusion, which leads to model bias towards high-value features and limits fusion accuracy. Second, when attempting to capture dynamic features under fluctuating production conditions, these methods typically rely on overly complex hierarchical architectures, imposing severe computational overheads that are unsuitable for real-time industrial edge deployment [28].

Regarding dynamic scene adaptation, methods employing DBNs, improved particle filters, or EMD-ANFIS have shown strong performance in non-stationary environments [29,30,31]. Yet, these approaches generally treat dynamic changes as random continuous drifts. Engine testing, conversely, involves discrete, deterministic phase transitions (e.g., Start-up to High-speed). Existing methods lack rapid response mechanisms for such abrupt state switching, resulting in prediction lags.

To bridge these operational gaps—specifically addressing scale inconsistency, edge-computing latency, and deterministic regime switching—the proposed CSWE and ARSR strategies are specifically designed to handle localized classification boundaries and phase-adaptive dynamic tracking.

## 3. Methodology

### 3.1. Overall Framework

**Figure 1:**
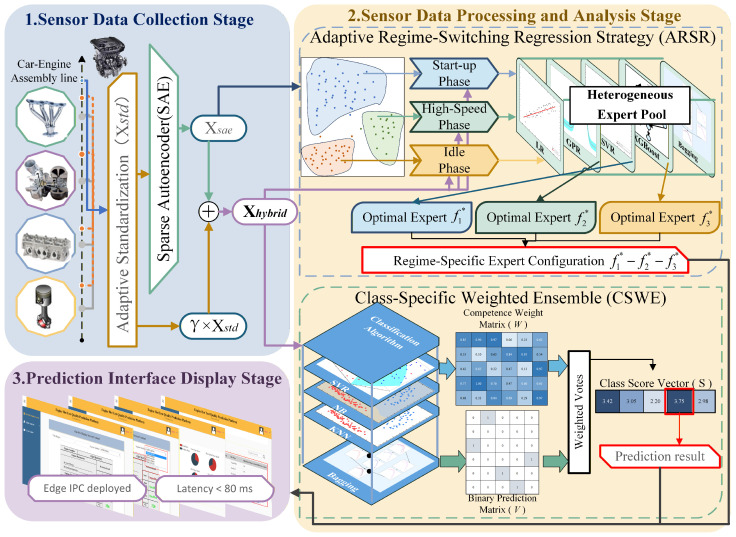
Overall structure of the proposed framework. The arrows indicate the direction of data processing and information flow across different stages and modules.

In this section, we detail a unified diagnostic and predictive framework specifically engineered for the high-precision requirements of automotive engine assembly lines. As illustrated in Figure 1, the architecture is designed to overcome the inherent challenges of industrial cold-test environments, such as heterogeneous sensor noise and non-stationary operational regimes, through a logically cohesive pipeline.

The pipeline initiates by mapping high-dimensional, multi-modal sensor streams—including dynamic torque, fluid pressure, and rotational speed—into a robust latent space. To mitigate the curse of dimensionality while preserving critical physical transients, we implement a Sparse Autoencoder (SAE) augmented with a noise-aware residual fusion mechanism. This dual-path feature extraction architecture generates unified hybrid features that successfully leverage both deep latent fault patterns and raw physical characteristics.

Building upon this robust feature representation, the framework bifurcates into two specialized algorithmic pathways to execute critical quality gate functions. For continuous performance tracking, we propose an Adaptive Regime-Switching Regression (ARSR) strategy. This regression module employs a divide-and-conquer paradigm to dynamically assign specialized local experts to distinct non-linear operating regimes, specifically the start-up, idle, and high-speed phases. Concurrently, to address the discrete defect detection task, we introduce a Class-Specific Weighted Ensemble (CSWE) strategy. By utilizing a competence-based weighting mechanism, this classification branch effectively navigates the extreme class imbalance of rare engine defects and optimizes the interception of highly variable failure modes.

Ultimately, the framework bridges the gap between algorithmic inference and industrial operation through an edge-deployed prediction interface. Hosted on line-side industrial PCs, the system provides real-time feedback to assembly line operators via a comprehensive human-machine interface (HMI). Operating with an optimized average inference latency of under 80 ms, this deployment supports instantaneous go/no-go quality decisions while enabling long-term defect tracing based on unique engine identification numbers to facilitate root cause analysis.

### 3.2. Feature Extraction

Due to the high dimensionality and scale inconsistencies of heterogeneous multi-sensor data, direct supervised learning is prone to overfitting and gradient instability. To address this, we propose a robust two-stage feature extraction pipeline: an Adaptive Standardization Mechanism for scale unification and a Sparse Autoencoder (SAE) augmented with Noise-Aware Residual Fusion for non-linear feature compression.

#### 3.2.1. Data Standardization

**Figure 2:**
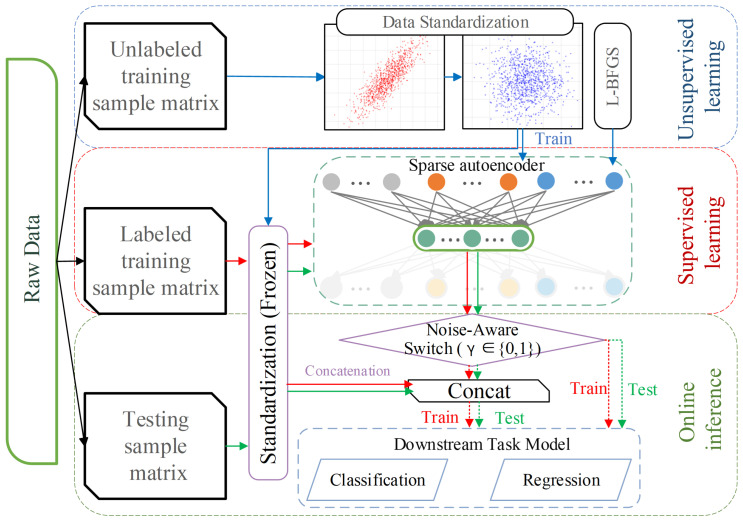
The proposed three-stage semi-supervised learning framework.The blue arrows denote the unsupervised pre-training stage, which exclusively utilizes unlabeled data. The red arrows represent the supervised training pathway, wherein labeled data are employed to train the downstream model. The green arrows illustrate the flow of test samples during the online inference phase. (**Top**) Unsupervised Learning Stage: Standardization statistics and SAE weights are learned exclusively from unlabeled process data to prevent leakage. (**Middle**) Supervised Training Stage: Labeled samples are processed via the frozen standardization module. A Noise-Aware Switch (γ) controls the residual connection, adaptively fusing deep latent features with raw signals to train the downstream predictors. (**Bottom**) Online Inference Stage: New test samples undergo the identical frozen pipeline for end-to-end diagnosis.

To guarantee an unbiased assessment and prevent data leakage, we implemented a strict data isolation protocol. The overall semi-supervised learning workflow, including the Noise-Aware Residual Fusion mechanism, is systematically illustrated in Figure 2.

To eliminate scale inconsistencies among heterogeneous sensors and ensure numerical stability for the SAE, we enforce a strict standardization protocol before feature learning. As illustrated in the pipeline, this module standardizes the input matrix X.

Given the varying degrees of feature correlation in different datasets, the standardization implementation is configured as follows: For the industrial sensor data, which exhibits severe multicollinearity (e.g., strong coupling between torque and pressure), we employ PCA Whitening. This transformation, defined as

(1) $$X_{white}=\left(X-\mu \right)U{\left(\Lambda +\epsilon I\right)}^{-1/2},$$

simultaneously standardizes the variance and decouples feature correlations to provide an isotropic input space. For benchmark datasets where feature independence is relatively high, we simplify the process to standard Z-Score or Min-Max scaling. This maps inputs to the sensitive region of the activation function (e.g., [0,1]) while preserving the original feature manifold.

To prevent data leakage, all statistics (e.g., mean μ, eigenvector matrix U) are computed *solely* from the unlabeled training set and strictly applied to the validation and test sets.

#### 3.2.2. Sparse Autoencoder with Noise-Aware Residual Fusion

Following standardization, a Sparse Autoencoder (SAE) is employed to extract latent patterns. As illustrated in Figure 2, the network compresses the high-dimensional input $N_{in}$ into a compact feature vector $N_{hidden}$ ($N_{hidden}\ll N_{in}$).

Unlike standard Autoencoders, we enforce a sparsity constraint to suppress background instrumentation noise. The network parameters *W* and *b* are optimized by minimizing a composite cost function $J_{sparse}$:

(2) $$J_{sparse}\left(W,b\right)=\underset{J_{MSE}}{\underset{︸}{\frac{1}{m}\sum_{i=1}^{m}\frac{1}{2}{∥h_{W,b}\left(x^{\left(i\right)}\right)-x^{\left(i\right)}∥}^{2}}}+\underset{\Omega_{weights}}{\underset{︸}{\frac{\lambda }{2}\sum_{l=1}^{2}{∥W^{\left(l\right)}∥}_{F}^{2}}}+\underset{\Omega_{sparsity}}{\underset{︸}{\beta \sum_{j=1}^{s}\mathrm{KL}\left(\rho ∥{\hat{\rho }}_{j}\right)}},$$

where the three terms represent the reconstruction fidelity (MSE), the L2 weight decay (weighted by λ), and the sparsity penalty (weighted by β), respectively. The Kullback–Leibler (KL) divergence constrains the average activation ${\hat{\rho }}_{j}$ to a low sparsity target ρ, effectively filtering out non-salient noise artifacts.

To ensure fast convergence on resource-constrained edge devices, we utilize the L-BFGS algorithm (Limited-memory Broyden–Fletcher–Goldfarb–Shanno). This quasi-Newton method approximates the inverse Hessian matrix to achieve superlinear convergence, making it superior to standard SGD for high-dimensional industrial optimization.

While SAEs are effective at extracting non-linear patterns, the compression process may inevitably filter out high-frequency physical details. To address this, we introduce a conditional Residual Feature Fusion mechanism. To ensure comprehensive representation, the hybrid feature vector is constructed as:

(3) $$X_{hybrid}=\mathrm{Concat}\left(\gamma \cdot X_{std},X_{sae}\right),$$

where γ∈{0,1} is a dual-purpose gating parameter determined by both the signal-to-noise ratio (SNR) and the intrinsic dimensionality ($N_{in}$) of the input space. $X_{sae}$ represents the deep latent features extracted by the SAE, and $X_{std}$ denotes the standardized physical signals.

- Mode I: Compression & Denoising (γ=0). For the industrial dataset, which is characterized by extremely high dimensionality ($N_{in}\approx$ 10,000) and significant noise, we strictly set γ=0. This setting enforces the dimensionality reduction capability of the SAE (10,000 $\to N_{hidden}$), preventing the “curse of dimensionality” and overfitting while filtering out background interference.
- Mode II: Information Maximization (γ=1). For standard benchmarks (e.g., CMAPSS) with low dimensionality ($N_{in}<50$) and high signal fidelity, we set γ=1. In this low-dimensional regime, overfitting is not the primary constraint. Thus, the residual connection allows the model to leverage both the deep abstract features and the raw physical signals, maximizing predictive precision.

### 3.3. Quality Verification and Performance Prediction Modules

#### 3.3.1. Adaptive Regime-Switching Regression Strategy

To address the non-stationary multi-stage patterns inherent in complex mechanical equipment, we propose an Adaptive Regime-Switching Regression Strategy (ARSR). This framework discards the reliance on a single monolithic model; instead, it decomposes the prediction task into local sub-problems across multiple stages, automatically identifying operating regimes and assigning the most adaptive “Expert Algorithm” to each.

**Figure 3:**
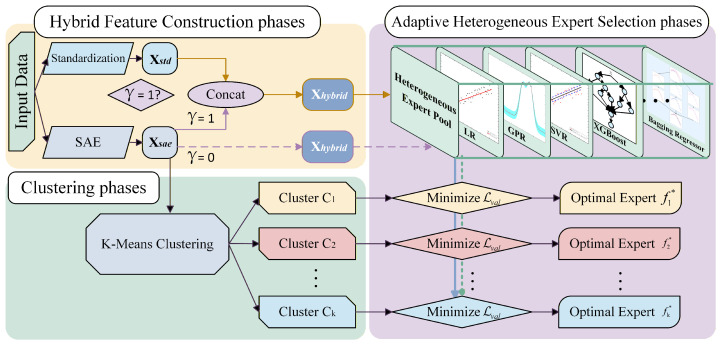
Framework of the Adaptive Regime-Switching Regression Strategy (ARSR).

The workflow consists of three tightly coupled phases, as illustrated in Figure 3.

In the Hybrid Feature Integration Phase, the ARSR module directly ingests the residual-fused features $X_{hybrid}$ generated from Stage 1 (Section 3.2). This comprehensive hybrid representation ensures that the downstream regression experts are provided with a panoramic view of the equipment’s dynamic status, capturing both explicit physical magnitudes ($X_{std}$) and implicit non-linear correlations ($X_{sae}$).

In the Clustering Phase, to address the challenge of unsupervised structure discovery in industrial regression scenarios, we utilize K-Means clustering to uncover the intrinsic data structure. We apply K-Means clustering specifically to the latent feature subspace ($X_{sae}$) of the hybrid representation to partition the data into *K* independent operating regime sets $R=\{C_{1},C_{2},\ldots ,C_{K}\}$.

In the Adaptive Heterogeneous Expert Selection Phase, since different models exhibit varying performance across different data distributions, we construct a heterogeneous expert pool $H_{pool}$ containing algorithms with diverse inductive biases (e.g., LR, SVR, XGBoost, GBR). For each identified cluster $C_{k}$, the system performs an internal competition. The data within $C_{k}$ is split into training and validation sets. The optimal local expert $f_{k}^{*}$ is selected by minimizing the validation error:

(4) $$f_{k}^{*}=\underset{f\in H_{pool}}{arg min}\left(L_{val}\left(f,D_{C_{k}}\right)\right),$$

where $f_{k}^{*}$ denotes the optimal expert selected for regime $C_{k}$. The selection is performed by iterating through every candidate model *f* within the heterogeneous pool $H_{pool}$ and identifying the one that minimizes the validation loss $L_{val}$ on the regime-specific validation set $D_{C_{k}}$.

During the inference phase, a query sample is first assigned to a cluster via the nearest cluster centers, and the final prediction is generated by the corresponding optimal expert.

#### 3.3.2. Class-Specific Weighted Ensemble Classification

Parallel to the regression task, the End-of-Line (EOL) defect detection (classification) relies on the same hybrid features ($X_{hybrid}$) extracted from Stage 1. However, relying on a single global classifier is risky due to the distinct decision boundaries of different defect types. For instance, distance-based models (e.g., KNN) may excel at clustering geometric faults, while probability-based models (e.g., Naive Bayes) might perform better on statistical anomalies. Standard majority voting overlooks these specific competencies and tends to bias toward the majority class in imbalanced industrial datasets.

To resolve this, we propose a Class-Specific Weighted Ensemble (CSWE) strategy. Instead of assigning a uniform weight to a classifier globally, we dynamically assign voting weights based on each classifier’s historical validation performance for specific defect categories.

**Figure 4:**
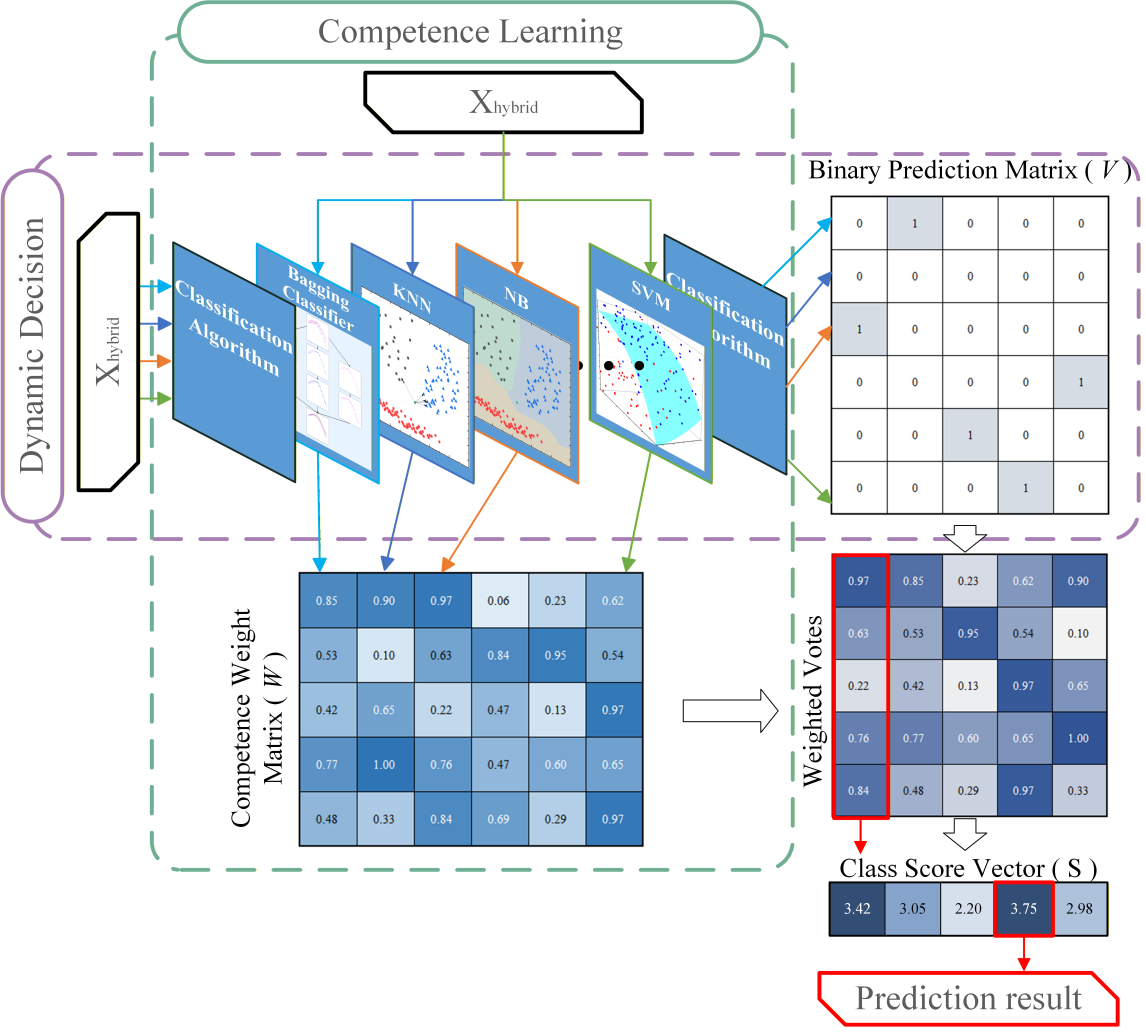
Schematic of the Class-Specific Weighted Ensemble (CSWE) strategy. In the figure, the colored thin arrows (excluding the red one) represent the individual output results of different base classifiers, while the red arrow indicates the final classification prediction of the system. The process is divided into two phases: (1) Competence Learning, where the weight matrix *W* is constructed offline based on validation F1-scores; and (2) Dynamic Decision, where weights are retrieved online to compute the Class Score Vector *S* for final prediction.

The implementation of CSWE consists of two logically coupled phases:

In the Competence Learning Phase, we construct the expert knowledge base by integrating multiple heterogeneous algorithms (e.g., SVM, KNN, Gaussian Naive Bayes, and Bagging). Let *m* be the number of algorithms and *n* denote the total number of target defect classes. We construct the Competence Weight Matrix $W\in R^{m\times n}$, defined as:

(5) $$W=\left[\begin{array}{cccc} w_{1,1} & w_{1,2} & \cdots & w_{1,n}\\ w_{2,1} & w_{2,2} & \cdots & w_{2,n}\\ \vdots & \vdots & \ddots & \vdots \\ w_{m,1} & w_{m,2} & \cdots & w_{m,n} \end{array}\right],$$

where each row ${\vec{w}}_{j}$ represents the performance profile of the *j*-th classifier. To populate this matrix, we utilize the Class-Specific F1-Score on the validation set:

(6) $$w_{j,k}={F1}_{j,k}=\frac{2\cdot P_{j,k}\cdot R_{j,k}}{P_{j,k}+R_{j,k}}+\epsilon ,$$

where ϵ (typically $10^{-7}$) ensures numerical stability. This matrix *W* (shown in the bottom-left of Figure 4) is frozen after learning.

In the Dynamic Decision Phase, when a query sample (represented by the hybrid feature vector $X_{hybrid}$) is input into the system, the decision process follows three steps:

1. Binary Prediction: The classifiers generate a Binary Prediction Matrix $V\in {\left\{0,1\right\}}^{m\times n}$ (Top-right of Figure 4), where $v_{j,k}=1$ indicates model *j* predicts class *k*.
2. Weighted Voting: The system computes the Weighted Votes by taking the element-wise product of the dynamic binary predictions (*V*) and the static competence weights (*W*).
3. Score Aggregation: Finally, we calculate the Class Score Vector $S\in R^{n}$ by summing the weighted votes column-wise:
  (7) $$S_{k}=\sum_{j=1}^{m}w_{j,k}\cdot v_{j,k},\;\forall k\in \left[1,n\right].$$

The final prediction $c^{*}$ is the index corresponding to the maximum value in the score vector *S*:

(8) $$c^{*}=\underset{k}{arg max}\left(S_{k}\right).$$

## 4. Data Description and Experimental Setup

### 4.1. Industrial Dataset Construction and Preprocessing

The industrial dataset utilized in this study is derived from the H4 assembly line of a leading automotive manufacturer, specifically focusing on the JL478QEA-AJ engine model. To ensure comprehensive monitoring, we implemented a structured retrieval protocol within the Engine Test Data Management Database. By adopting the unique engine identification number (Engine ID) as the primary association key, we integrated heterogeneous records across three distinct physical domains: Process Data, Quality Data, and Performance Data.

The collected industrial dataset is characterized by extreme high-dimensionality ($N_{total}>$ 12,000) and multi-source heterogeneity, which is physically mapped to a distributed sensor suite across the H4 assembly line following the hierarchical sequence from OP10 to OP130. As illustrated in Table 1, the data is organized into a three-tier hierarchical structure.

Specifically, the Process Data (N> 11,000) is primarily derived from high-frequency torque and angle encoders at fastening stations (e.g., OP10–OP90), where continuous mechanical traces are recorded at a uniform sampling frequency of 5 Hz to capture transient assembly dynamics. The Quality Data (N≈1100) originates from specialized pressure transducers and flow meters at intermediate testing units, such as OP60 for leakage tests and OP100 for fuel-rail integrity. Finally, the Performance Data comprises 29 functional metrics (e.g., engine speed and torque) collected via high-precision tachometers and dynamometers at the End-of-Line (EOL) hot-test bench (OP130). This mapping ensures that the high-dimensional feature space accurately reflects the latent physical states of the entire manufacturing trajectory.

During the data extraction process, records were pulled from the database utilizing the inspection time and the unique Engine ID as query keys. For each engine sample, the retrieved attributes encompass the Engine ID, inspection time, hierarchical data categories (which encode the specific workstation and process stage), specific inspection items, and their corresponding numerical values. To accurately reconstruct the chronological assembly trajectory, the raw data was strictly sorted based on a hierarchical priority: Engine ID → Category (Workstation) → Inspection Time. An illustrative example of this exported data structure is presented in Table 2.

Once the raw chronological trajectories were reconstructed, the next critical step was formulating the predictive modeling tasks by assigning reliable ground truth labels. This was achieved through a rigorous dual-verification process. Primary labels (e.g., Pass/Fail for defect classification, and continuous RPM/Torque values for regression) were automatically recorded by the certified EOL test bench. Furthermore, to address potential labeling noise near decision boundaries, a specific subset of samples underwent manual re-inspection by senior quality engineers.

Following label assignment, the raw sensor streams underwent a rigorous preprocessing pipeline. This process consists of four synchronized stages: (1) Outlier Removal to filter discrete anomalies; (2) Gap Imputation using numerical interpolation; (3) Noise Reduction via smoothing filters; and (4) Standardization to normalize physical scales. To guarantee data quality, a strict sparsity threshold was enforced: any sensor channel or sample instance exhibiting a missing data rate exceeding 30% was discarded. The justification for this specific threshold is further analyzed in Section 6.

**Figure 5:**
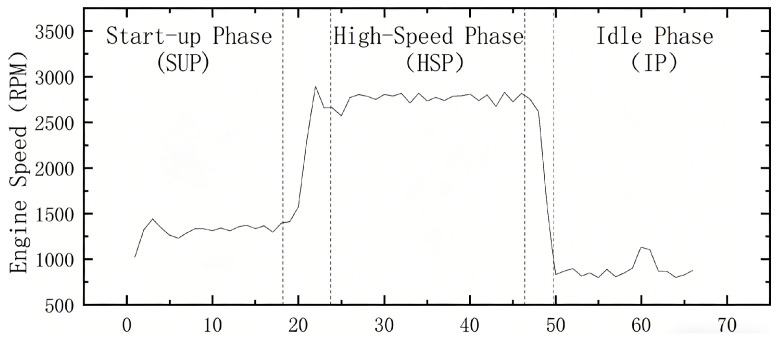
Representative engine speed profile illustrating the deterministic phase segmentation. The solid line represents the continuous engine speed, while the vertical dotted lines indicate the boundaries separating the designated operational phases (SUP, HSP, and IP) and the excluded transient states.

Before quantifying the final dataset for model training, the continuous engine test cycle must be segmented to align with the proposed regime-switching architecture. As shown in Figure 5, the dynamic operational profile is deterministically partitioned into three fundamental phases based on RPM thresholds and thermodynamic states: (1) the Start-up Phase (SUP), characterized by a rapid, linear transient acceleration; (2) the High-Speed Phase (HSP), exhibiting complex, non-linear high-frequency vibrations; and (3) the Idle Phase (IP), representing low-amplitude, steady-state dynamics.The transient states between these conditions were excluded to minimize non-stationary switching noise.

Consequently, the final experimental dataset distribution is independently quantified across these three operational phases, as detailed in Table 3. To prevent data leakage during model evaluation, strict stratification by engine serial number was enforced, ensuring that samples from the exact same physical engine do not appear simultaneously in the training and testing sets.

### 4.2. Public Benchmark Datasets

While the proprietary industrial dataset described in Section 4.1 establishes a rigorous experimental environment with explicit physical constraints and deterministic operational phases, relying exclusively on it limits the assessment of the framework’s broader applicability. To ensure a comprehensive evaluation of its generalization capability—particularly concerning latent regime discovery, extreme class imbalance, and high-dimensional noise—we extended the experimental setup to include three widely recognized public benchmark datasets.

Rather than evaluating the framework holistically on a single dataset, these public benchmarks were strategically selected to isolate and stress-test specific algorithmic components under diverse industrial constraints. As summarized in Table 4, the selected datasets introduce critical data challenges that are pervasive in complex manufacturing environments, serving as targeted testbeds for both the defect detection (classification) and performance tracking (regression) modules.

### 4.3. Experimental Setup and Evaluation Protocol

To ensure both computational efficiency during training and practical viability during deployment, the experiments were conducted across two distinct hardware platforms. Model training, public benchmark evaluations, and ablation studies were executed on a high-performance server equipped with an Intel Xeon Gold 6430 CPU and an NVIDIA RTX 4090 GPU (24 GB VRAM), ensuring the rapid convergence of complex deep learning architectures. Conversely, the real-world industrial validation was deployed on the actual line-side edge computing cluster powered by legacy Intel Xeon E5-2680 v4 processors, explicitly validating the framework’s low-latency inference capabilities in resource-constrained manufacturing environments. The software stack was standardized on Python 3.8, utilizing PyTorch 2.0.0 for deep neural networks (SAE, Transformer, CNN) and Scikit-learn 1.0 for traditional machine learning models.

To rigorously evaluate the superiority of the proposed framework within this environment, we selected a comprehensive set of state-of-the-art and conventional baseline models. As summarized in Table 5, these comparative methods are systematically categorized into ensemble learning, deep learning, and traditional algorithms, each serving a specific evaluative purpose in addressing high-dimensional industrial data.

To provide a comprehensive and statistically robust assessment of model performance, we report the Mean ± Standard Deviation of the evaluation metrics summarized in Table 6. To ensure strict reproducibility, the global random seed was fixed at 42. The reported results represent the average of 10 independent runs for the classification tasks and the AI4I tool wear dataset. Due to the high computational cost associated with lengthy time-series sequences, results for the NASA C-MAPSS dataset were averaged over 5 independent runs.

Regarding task-specific evaluation, the classification analysis (defect detection) strategically prioritizes maximizing Recall (to strictly prevent defective engines from escaping to the market) and the F1-Score (to ensure balanced assessment under extreme class imbalance). For the regression task (performance tracking), we utilize Mean Squared Error (MSE), the Coefficient of Determination ($R^{2}$), and Mean Absolute Percentage Error (MAPE). This combination precisely captures the model’s sensitivity to physical outliers, validates the goodness-of-fit to underlying thermodynamic trends, and interprets scale-independent relative errors across vastly different RPM regimes (e.g., Start-up versus Idle phases).

## 5. Experimental Results

We present a comprehensive quantitative evaluation of the proposed framework. The experimental validation is structured into three parts: First, we assess the framework’s generalization capability and state-of-the-art (SOTA) competitiveness on widely recognized public benchmarks (Section 5.1). Second, we conduct an ablation study to analyze the contribution of core components such as noise-aware residual fusion (Section 5.2). Finally, we demonstrate the framework’s practical engineering performance in the real-world automotive assembly line (Section 5.3).

### 5.1. Generalization Performance on Public Benchmarks

To verify the robustness of the proposed method against challenges such as high-dimensional noise, class imbalance, and latent operating regimes, we compared our framework with state-of-the-art baselines, including Random Forest, XGBoost, LightGBM, CNN, and Transformer. The comparison encompasses traditional machine learning models (Random Forest, SVM), ensemble methods (XGBoost, LightGBM), and deep learning architectures (CNN, Transformer).

#### 5.1.1. Defect Detection Performance (Classification)

For the classification tasks on the AI4I 2020 (Imbalance Challenge) and UCI SECOM (High-Dimensionality Challenge) datasets, the primary objective is to evaluate whether the proposed Class-Specific Weighted Ensemble (CSWE) can identify minority class failure samples more effectively than traditional methods.

**Figure 6:**
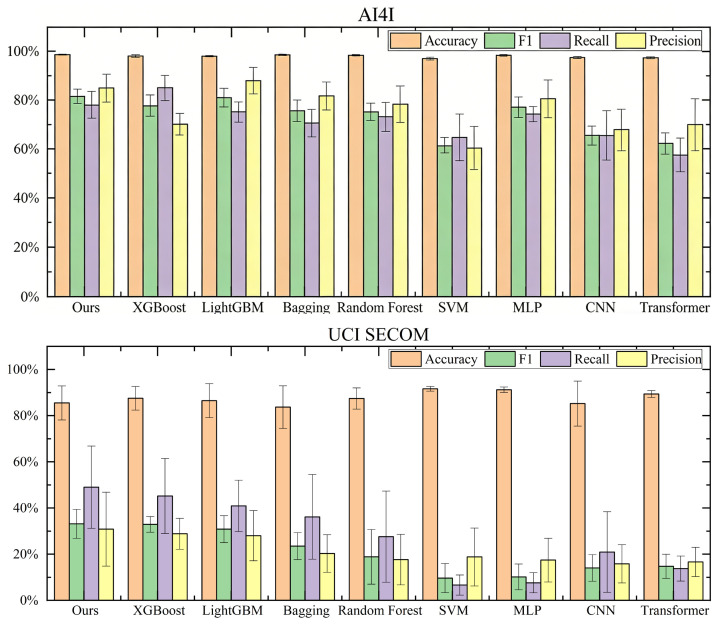
Performance comparison on AI4I 2020 and UCI SECOM datasets.

Figure 6 illustrates the comparative performance across different algorithms. Table 7 details the quantitative metrics.

On the AI4I 2020 dataset, the proposed method achieved the highest F1-Score (81.55%), slightly outperforming the second-best model (LightGBM, 81.05%) and significantly exceeding XGBoost (77.22%). While XGBoost exhibited the highest Recall (85.74%), its Precision (70.29%) was considerably lower than ours, indicating a higher rate of false alarms. In contrast, our method achieved a more robust and balanced performance, maintaining a high Precision (85.70%) while sustaining strong recall. This balance is critical in industrial manufacturing to prevent the unnecessary downtime and costs associated with false positive failure predictions.

On the UCI SECOM dataset, which represents an extreme case of high dimensionality and noise, deep learning models such as Transformer and CNN struggled to converge, yielding extremely low F1-Scores. Our method, leveraging the noise-aware SAE for feature extraction, achieved the best Recall (49.05%) and F1-Score (33.17%), demonstrating superior robustness in sparse, noisy feature spaces.

#### 5.1.2. Degradation Prediction Performance (Regression)

**Figure 7:**
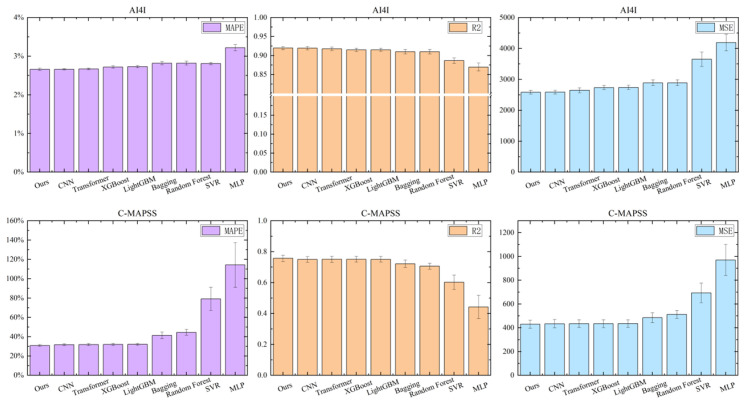
Performance comparison on AI4I Tool Wear and NASA C-MAPSS FD002 datasets.

For regression tasks, we evaluated the framework on the AI4I 2020 (Tool Wear) and NASA C-MAPSS FD002 (Remaining Useful Life prediction) datasets, with results shown in Figure 7. We selected the more complex FD002 subset as a key benchmark, as its core challenge lies in the presence of six distinct operating conditions. We compared performance using Mean Squared Error (MSE), $R^{2}$, and Mean Absolute Percentage Error (MAPE), as detailed in Table 8.

For the AI4I Tool Wear task, our method achieved an MSE of 2584.88, slightly outperforming the 1D-CNN (2589.83) and significantly beating traditional regressors such as SVR (3651.86). The low standard deviation across metrics further evidences the consistency of the results.

On the NASA C-MAPSS FD002 dataset, tabular Transformer and MLP models failed to model the complex multi-regime manifold, resulting in high MSE values (970.55 and 692.97, respectively). In contrast, our framework achieved the lowest MSE of 424.49, surpassing the best baseline (Bagging, MSE = 433.81) and XGBoost (MSE = 434.01). This demonstrates the effectiveness of the adaptive regime-switching strategy, which dynamically assigns optimal local experts to distinct latent regimes.

### 5.2. Ablation Study: Impact of Key Components

To rigorously evaluate the contribution of each module within the proposed framework, we conducted an ablation study on the UCI SECOM (Classification) and NASA FD002 (Regression) datasets. We compared the full model against three variants:

1. w/o Sparsity (AE): Replaces the Sparse Autoencoder with a standard dense Autoencoder, removing the ρ penalty in the loss function.
2. w/o SAE (Raw): Removes the feature extraction module entirely, feeding raw standardized sensor data directly into the ensemble models.
3. w/o Partitioning: Removes the K-Means clustering and dynamic switching mechanism, training a single global expert pool on the entire dataset.

The quantitative results are summarized in Table 9.

On the UCI SECOM dataset, the “w/o SAE (Raw)” variant achieved the highest Accuracy (88.60%) but suffered from a significantly lower Recall (40.48%) compared to the Full Model (49.05%). This phenomenon occurs because the high-dimensional noise in raw features causes the model to bias towards the majority class (non-defective), leading to missed detections.

Furthermore, removing the sparsity constraint (“w/o Sparsity”) caused the F1-Score to drop drastically from 33.17% to 23.94%. This validates that the Sparse Autoencoder is not merely performing dimensionality reduction, but is actively filtering out incoherent sensor noise through the sparsity penalty, which is crucial for identifying latent fault patterns in complex manufacturing data.

The impact of the Adaptive Regime-Switching Regression (ARSR) Strategy is evident in the NASA FD002 regression task. When the clustering-based partitioning was removed (“w/o Partitioning”), the model was forced to fit a single global function to the six distinct operating conditions, resulting in a degradation of MSE from 424.49 to 474.45. The Full Model, by “dividing and conquering” the latent regimes, effectively minimized the prediction error, confirming the necessity of the multi-expert architecture for complex industrial processes.

### 5.3. Industrial Field Performance (Edge Deployment)

Following the theoretical validation on public benchmarks, the proposed framework was deployed on the JL478QEA-AJ automotive engine assembly line to evaluate its engineering viability.

To satisfy the strict real-time constraints (<100 ms Takt time) and limited computational resources of the line-side edge devices (Intel Xeon E5), we implemented two strategic optimizations. First, we replaced the unsupervised K-Means clustering within the ARSR module with deterministic, physics-based RPM thresholds (Start-up, High-speed, and Idle). This eliminates iterative computational overhead while ensuring entirely consistent regime assignment. Second, to mitigate memory bandwidth bottlenecks caused by the massive raw data dimensionality (>10,000), we operated the feature extraction module strictly in Mode I (γ=0). By utilizing only the compact SAE latent representations for downstream inference, the framework drastically reduces the feature footprint while intrinsically filtering high-frequency instrumentation noise.

#### 5.3.1. Task I: Defect Classification

The industrial classification dataset comprises 2090 unlabeled samples for SAE pre-training and 520 labeled samples for supervised validation. We evaluated the Class-Specific Weighted Ensemble (CSWE)—here instantiated as a Region-Weighted Ensemble to handle phase-specific defect patterns—against standard base classifiers and Bagging ensembles.

**Figure 8:**
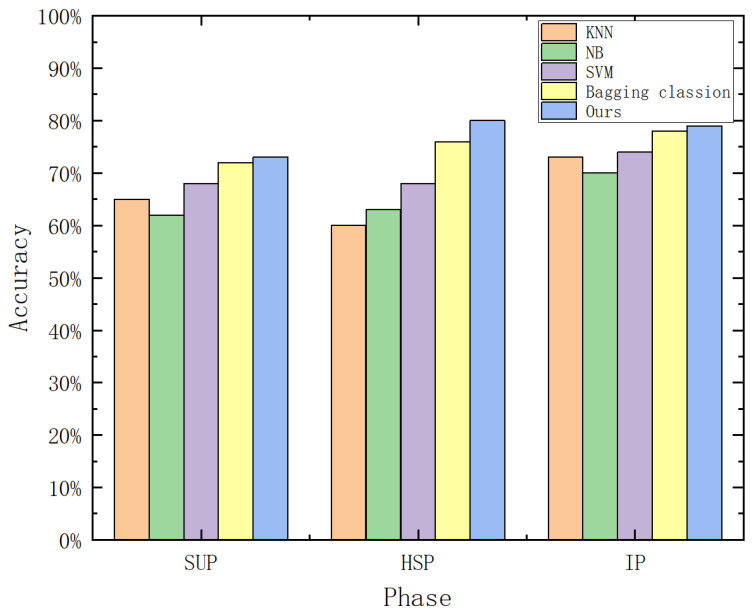
Accuracy comparison of engine speed qualification prediction across operating phases.

Figure 8 illustrates the accuracy comparison. The proposed method consistently outperforms baseline approaches, particularly in the High-speed Phase (HSP), where it maintains a stability margin of approximately 5% over competing methods.

Table 10 presents the quantitative metrics. While the improvement in Precision is modest (+1.63%), the most significant gain is observed in the Recall rate, which surged from 69.20% (Bagging) to 76.92% (Ours). In the context of industrial Quality Assurance (QA), Recall is the most critical metric as it represents the system’s ability to intercept defective engines. An improvement of 7.7% implies a substantial reduction in the risk of defective products escaping to the market.

#### 5.3.2. Task II: Continuous Performance Regression

It should be clarified that the regression target is not the engine speed as a variable of driver demand, but rather the engine’s functional response under a fixed test-bench load protocol. By predicting the expected mean speed and its standard deviation, the framework establishes a dynamic baseline for ‘normal’ behavior. Consequently, any significant deviation from these predicted values serves as a micro-indicator to identify latent mechanical friction anomalies or ECU calibration faults that might otherwise remain undetected during standard steady-state monitoring.

In this task, the ARSR module dynamically assigned the optimal expert model to each physical phase based on their distinct data manifolds.

**Figure 9:**
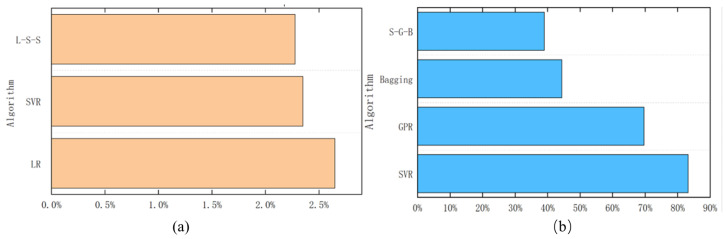
Performance comparison of regression algorithms: (**a**) Mean Engine Speed prediction (orange bars); (**b**) Engine Speed STD prediction (blue bars).

For the Mean Engine Speed prediction task, as illustrated in Figure 9a, the framework automatically selected Linear Regression (LR) for the linear Start-up phase, and Support Vector Regression (SVR) for the highly non-linear High-speed and Idle phases. This dynamic switching strategy (L-S-S) achieved a comprehensive MAPE of 2.276%, outperforming both standalone LR (2.646%) and SVR (2.350%). This confirms that the regime-based switching mechanism effectively captures the varying linearity of the engine’s thermodynamic speed curve.

Conversely, Figure 9b presents the prediction results for the Engine Speed Standard Deviation (STD), which acts as a micro-indicator for transient stability. Compared to global baseline models like Bagging (44.33%) and GPR (69.67%), our proposed phase-adaptive S-G-B combination (SVR-GPR-Bagging) achieved the lowest overall MAPE of 39.00%.

Table 11 summarizes the performance metrics. We observe notably higher MAPE values during the Idle Phase (IP), particularly for Speed STD. This is an inherent artifact of the metric rather than model failure: since the ground truth STD values in the idle phase approach zero, small absolute errors result in disproportionately large percentage errors. Despite this numerical sensitivity, the absolute precision remains within acceptable industrial tolerances.

It is important to address the seemingly high MAPE values in the STD prediction. During the Idle Phase, the ground-truth STD values are numerically minute (approaching absolute zero). Due to the mathematical formulation of the MAPE metric, dividing by such infinitesimal denominators inherently inflates the percentage errors, even for negligible absolute deviations. From a strict engineering perspective, the absolute prediction error during this phase remains well within the industry-standard tolerance limits (±10 RPM). This validates the system’s operational reliability and its capability to accurately distinguish between normal mechanical vibration noise and actual transient stability defects.

#### 5.3.3. System Efficiency and Economic Impact

The optimized framework was deployed on the edge computing cluster. By adopting deterministic regime switching and setting γ=0 (Pure SAE Mode), the system achieved an average inference latency of 80 ± 3 ms per engine. By bypassing the massive matrix multiplications required by deep global architectures, this lightweight configuration securely maintained the processing time within the strict 100 ms Takt Time boundary.

**Figure 10:**
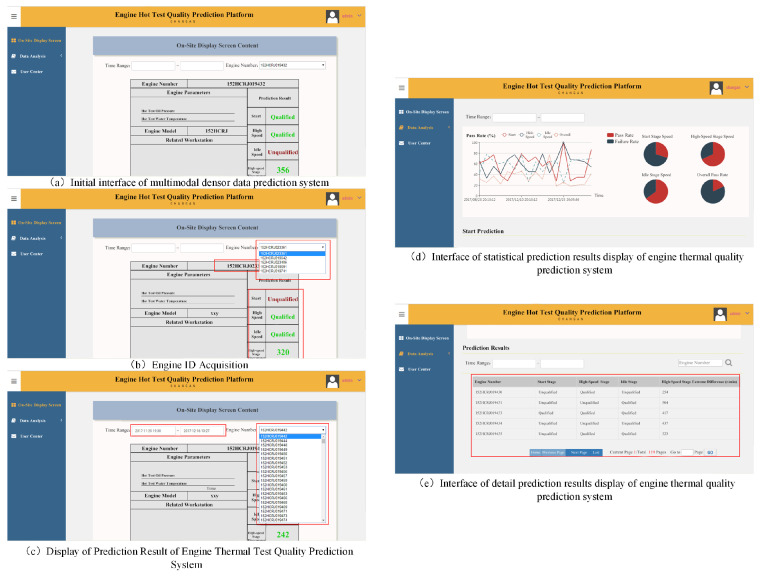
User Interface of the deployed Engine Quality Prediction System.

Following a three-month pilot run (interface shown in Figure 10), statistical analysis confirms that the engine repair rate decreased by 7.2%. This demonstrates that the edge-optimized “Lightweight” configuration of our framework successfully balances theoretical sophistication with engineering practicality.

## 6. Discussion

### 6.1. Mechanism Interpretability and Data Heuristics

**Figure 11:**
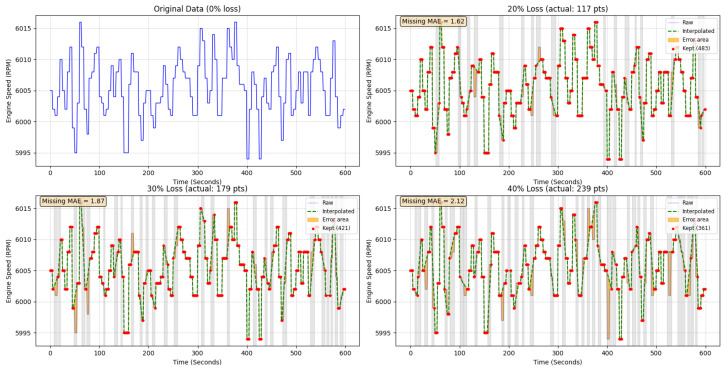
Waveform fragmentation and synthetic artifacts caused by numerical interpolation when missing data exceeds the 30% threshold. The solid blue line in the top-left panel represents the original raw engine speed data without any loss.

The selection of the 30% rejection threshold represents a deliberate trade-off between data fidelity and statistical representativeness, constrained by the 5 Hz sampling rate. At this frequency (0.2 s per interval), exceeding 30% loss in a workstation window results in continuous blind spots of 0.8–1.0 s. As demonstrated in Figure 11, since critical transient events—such as the 6000 RPM peak in performance tests—occur within a narrow 1.5–2.0 s window, such gaps lead to the complete erasure of original mechanical signatures.

The choice of 30% also prevents prohibitive data attrition. Setting a more stringent threshold (e.g., <10%) in a harsh industrial environment would lead to the rejection of over 40% of the captured samples. Such aggressive filtering not only restricts the training volume required for deep feature extraction but also introduces survivor bias, where the model only learns from “ideal” sensor conditions and fails to recognize rare defect patterns that often occur during unstable, noisy operational states.

The 30% boundary therefore serves as a optimal point. It maintains a sufficiently diverse training corpus while ensuring that the resulting synthetic artifacts remain within a manageable limit. While the SAE’s sparsity constraint (ρ) is highly effective at filtering random high-frequency instrumentation jitter, it is susceptible to the structured, overly-smooth curves generated by excessive interpolation. These artifacts mimic coherent physical patterns and can deceive the SAE into encoding “hallucinated” dynamics as valid latent features. By maintaining the 30% limit, we ensure that the SAE performs its primary role—extracting salient non-linear features and reducing dimensionality—without being contaminated by non-physical interpolated trends.

Furthermore, regarding the exclusion of transient conditions, it is essential to note the physical context of End-of-Line (EOL) testing. In industrial assembly, the steady-state phases (Start-up, High-speed, and Idle) serve as the primary indicators of assembly integrity and thermodynamic stability. Transient switching points often contain significant stochastic noise coupled with the test bench’s mechanical response rather than the engine’s intrinsic build quality. Therefore, focusing on these deterministic, stabilized regimes allows the framework to achieve more reliable defect interception by isolating high-fidelity quality signatures from environmental instrumentation artifacts.

### 6.2. Cost-Sensitive Diagnostics and Recall Prioritization

In the field of industrial quality assurance (QA), defect containment is of paramount importance. The financial penalty of a missed detection (False Negative) far outweighs the operational cost of a manual re-inspection triggered by a false alarm (False Positive). Missing even a minor micro-structural anomaly can lead to costly batch failures.

**Figure 12:**
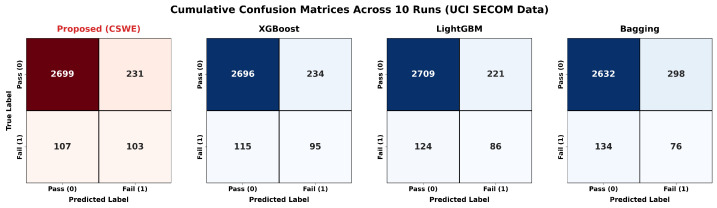
Cumulative confusion matrices across 10 independent runs on the UCI SECOM dataset. The proposed CSWE framework achieves the highest defect recall while simultaneously controlling false positives, outperforming global baselines in a defect-intolerant environment.

When confronted with extremely imbalanced and high-dimensional noise, traditional global baselines often adopt conservative decision boundaries that favor overall accuracy over minority class detection. As illustrated in the cumulative confusion matrices (Figure 12), baselines like LightGBM and Bagging successfully identified only 86 and 76 faults out of 210, respectively. While XGBoost performed better by intercepting 95 faults, it generated 234 false alarms.

In contrast, our proposed CSWE framework successfully recalled 103 true faults—achieving the highest detection rate—while simultaneously producing fewer false alarms (231 False Positives) than the strongest baseline. By leveraging SAE for robust feature extraction and localized routing, CSWE penetrates high-dimensional noise to establish a mathematically verified and practically deployable safety net: maximizing the capture of minority failure modes without inflating the manual inspection overhead.

**Figure 13:**
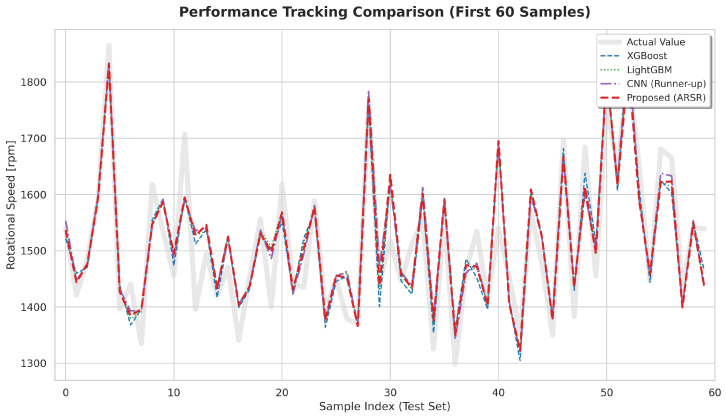
Comparative regression tracking of rotational speed on the AI4I 2020 dataset (first 60 test samples).

This philosophy of localized, adaptive routing is equally critical for continuous state monitoring. As shown in the regression tracking comparison (Figure 13) on the AI4I 2020 dataset, state-of-the-art global baselines already exhibit a strong capacity to capture the highly non-linear dynamics of rotational speed. While the ARSR module performs visually similarly to these powerful baselines, it maintains a subtly tighter adherence to the ground truth during rapid mechanical transients. This improved structural stability is quantitatively validated by a distinct reduction in Mean Squared Error (MSE) compared to all baselines. By more effectively suppressing the micro-transient errors (such as overshooting or undershooting) that could otherwise mask imminent mechanical instabilities, ARSR provides a more reliable continuous state estimation. Coupled together, CSWE’s robust defect interception and ARSR’s precise tracking establish a comprehensive, dual-layered safety guarantee for modern automated assembly lines.

### 6.3. Engineering Boundaries and Edge-Computing Viability

Transitioning from public benchmarks to industrial deployment requires context-aware optimization. Replacing computationally expensive K-Means clustering with explicit physics-based gating rules (predefined RPM thresholds) enables zero-latency regime assignment.

Crucially, edge-computing compatibility dictates the selection of downstream regression experts. While state-of-the-art Tabular Transformers exhibit formidable representational capacity, their global self-attention mechanisms inherently introduce a quadratic computational complexity $O(N^{2})$ with respect to feature dimensionality. On resource-constrained edge devices lacking dedicated GPU acceleration, accommodating such high FLOPs and memory bandwidth pressure is theoretically prohibitive and fundamentally incompatible with the strict Takt time constraints (<100 ms) of automated assembly lines. Our lightweight SAE-expert architecture resolves this bottleneck, maintaining an optimal Pareto balance between deep diagnostic capabilities and an ultra-low inference latency of 80 ± 3 ms.

Despite these optimizations, defining acceptable performance thresholds reveals the physical ceiling of single-modal tabular data. Industrial Quality Assurance rigorously prioritizes Recall (ideally > 95%) to prevent defective engines from entering the market. While our edge-deployed framework establishes a robust mathematically verified baseline with a 76.92% Recall and an 81.63% Precision, bridging the gap to near-absolute detection limits requires overcoming unrecorded manual variations and sensor base-noise. Future frameworks must transition to multi-modal sensory fusion, integrating Acoustic Emission (AE) sensors and real-time visual tracking to capture the microscopic structural anomalies that tabular data inherently fails to resolve.

## 7. Conclusions

In this study, we developed a robust diagnostic and predictive framework to address the inherent complexities of multi-sensor data in automotive engine assembly. By integrating a Sparse Autoencoder (SAE) for non-linear noise filtration with a Class-Specific Weighted Ensemble (CSWE) and an Adaptive Regime-Switching Regression (ARSR) strategy, the proposed approach effectively overcomes the persistent industrial challenges of extreme class imbalance and non-stationary operating dynamics.

Extensive experimental validation has demonstrated the profound algorithmic superiority of this methodology. For defect detection, the CSWE mechanism strategically prioritized minority failure modes, improving the defect interception recall rate by 7.72% over standard ensemble baselines, thereby minimizing the risk of defective engines escaping to the market. Simultaneously, for continuous performance tracking, the phase-adaptive ARSR strategy successfully decoupled the complex thermodynamic baseline, reducing the relative prediction error for transient stability indicators by approximately 12%.

Beyond theoretical accuracy, the framework proved highly efficient and economically valuable in a live production environment. By operating entirely on compact latent features and explicit physics-based routing rules, the edge-deployed system maintained an ultra-low average inference latency of 80±3 ms, strictly satisfying the line-side Takt time constraints. Furthermore, a three-month longitudinal pilot run validated its practical engineering impact, yielding a 7.2% reduction in overall engine repair rates and establishing a highly scalable paradigm for smart manufacturing quality assurance.

## 8. Limitations and Future Work

While the proposed framework demonstrates significant industrial applicability and establishes a robust baseline, we acknowledge several limitations that outline critical directions for future research.

First, and most fundamentally, relying exclusively on single-modal tabular process data (e.g., dynamic torque and fluid pressure) imposes a theoretical ceiling on defect detection precision. As addressed in our discussion, approaching a near-100% diagnostic accuracy limit is currently constrained by inherent sensor base-noise, microscopic tolerance stack-ups, and unrecorded manual operational variations. Therefore, a primary direction for future work is transitioning toward multi-modal sensory fusion. Integrating high-frequency Acoustic Emission (AE) sensors and real-time visual tracking data will be essential to capture the microscopic structural anomalies that low-frequency tabular data inherently fails to resolve.

Second, regarding regression evaluation metrics in low-amplitude regimes, the Mean Absolute Percentage Error (MAPE) exhibits extreme numerical sensitivity during the engine’s Idle phase, where ground-truth values for speed standard deviation approach zero. Although the relative performance of our regime-switching model remains superior, the inflated absolute MAPE values indicate that deterministic point-forecasting metrics may be inadequate for stability indices in near-zero scenarios. Future research will explore probabilistic forecasting and confidence interval analysis (e.g., Bayesian Neural Networks) to provide more robust, uncertainty-aware quantification in these low-amplitude operational zones.

Finally, the current framework operates on a static training paradigm, utilizing data collected during a specific production stabilization phase. However, real-world industrial environments are highly susceptible to concept drift caused by progressive tool wear, seasonal ambient temperature shifts, or supplier material batch changes. Currently, the model lacks a continuous online learning mechanism to dynamically adapt to these gradual distribution shifts. Future iterations will incorporate domain adaptation and online learning algorithmsto automatically fine-tune the decision boundaries in real-time. Additionally, validating the framework’s generalization capability across heterogeneous engine models and distinct assembly lines will be prioritized to establish a broader, universally applicable benchmark for industrial quality prediction.

## Figures and Tables

**Table 1:** Engine data categories and parameters.

Data Category	Category	No. of Params	Description
Process data	Basic data	54	Connecting rod bearing clearances, flatness, depth, camshaft clearances.
	Fastening torque data	11,381	Final torque, rotation angle, torque/angle ratio.
	Scaling application data	261	Width and offset for lower block, spark plug, front cover, oil pan.
Quality data	Cold test	1027	Short/Long cold test signals
	Leakage test	150	Leakage test results
Performance data	Performance data	29	Speed, temperatures, pressures, Electronic Control Unit (ECU) parameters

**Table 2:** Illustrative data structure of the exported engine sensing records. The populated entries (e.g., YY-MM-DD, XX1, a, b) serve as generic placeholders to demonstrate the underlying data schema and hierarchical sorting logic.

ID	TIME	CATEGORY		ITEM	VALUE
152HCRJ022703	YY-MM-DD hh:mm:01	XX1	XXX1	SS1	a
152HCRJ022703	YY-MM-DD hh:mm:02	XX1	XXX1	SS2	b
152HCRJ022703	YY-MM-DD hh:mm:03	XX1	XXX2	SS1	c
152HCRJ022703	YY-MM-DD hh:mm:04	XX1	XXX2	SS2	d
…	…	…	…	…	…

**Table 3:** Distribution of the experimental dataset across operational phases.

Algorithm	Unlabeled Samples	Labeled Samples	Stage
Adaptive Regime-Switching Regression Strategy (ARSR)	2090	520	SUP
	2090	520	HSP
	2090	520	IP
Class-Specific Weighted Ensemble (CSWE)	620	135	SUP
	620	135	HSP
	620	135	IP

**Table 4:** Summary of Public Benchmark Datasets and Key Industrial Challenges.

Dataset	Task Type	Samples	Features	Key Industrial Challenge
AI4I 2020 [32]	Classification	10,000	14	Severe class imbalance (∼3.4% failure rate)
	Regression			Continuous multivariate degradation tracking
UCI SECOM [33]	Classification	3140	591	“Curse of dimensionality”, high noise, sparsity
NASA C-MAPSS FD002 [34]	Regression	N/A	21	Six unlabelled, latent operating conditions

**Table 5:** Taxonomy of Baseline Methods for Comparative Evaluation.

Category	Algorithms	Rationale for Inclusion
Ensemble Learning	XGBoost, LightGBM, Random Forest	Considered the “gold standard” for tabular data in industrial applications; evaluates the framework’s capability in handling non-linear feature interactions and high-dimensional sparse data.
Deep Learning	Transformer, 1D-CNN, MLP	Assesses representation and sequence modeling capabilities: Transformer for capturing global dependencies, 1D-CNN for extracting local temporal features, and MLP as a standard neural network baseline.
Traditional Methods	SVM, Bagging	Established industrial benchmarks; utilized to explicitly quantify the performance gains achieved by advanced non-linear and regime-adaptive modeling.

**Table 6:** Summary of Evaluation Metrics for Classification and Regression Tasks.

Task Category	Metric	Formula/Definition
Classification	Accuracy	$\frac{TP+TN}{TP+TN+FP+FN}$
	Precision	$\frac{TP}{TP+FP}$
	Recall (Sensitivity)	$\frac{TP}{TP+FN}$
	F1-Score	$2\cdot \frac{Precision\cdot Recall}{Precision+Recall}$
Regression	Mean Squared Error (MSE)	$\frac{1}{n}\sum_{i=1}^{n}{\left(y_{i}-{\hat{y}}_{i}\right)}^{2}$
	Coefficient of Determination ($R^{2}$)	$1-\frac{\sum {\left(y_{i}-{\hat{y}}_{i}\right)}^{2}}{\sum {\left(y_{i}-\bar{y}\right)}^{2}}$
	Mean Abs. Percentage Error (MAPE)	$\frac{1}{n}\sum_{i=1}^{n}\left|\frac{y_{i}-{\hat{y}}_{i}}{y_{i}}\right|$

**Table 7:** Classification Performance on AI4I 2020 and UCI SECOM Datasets (Mean ± Std %). The best results are highlighted in **bold**, and the second-best are underlined.

Dataset	Model	Accuracy (%)	F1-Score (%)	Recall (%)	Precision (%)
AI4I 2020	Ours (SAE + CSWE)	98.80 ± 0.24	**81.55 ± 3.51**	77.94 ± 3.83	85.70 ± 4.89
	LightGBM	**98.81 ± 0.24**	81.05 ± 4.63	75.29 ± 7.05	**88.42 ± 4.20**
	Bagging	98.45 ± 0.28	75.51 ± 4.56	70.44 ± 5.71	81.77 ± 5.76
	Random Forest	98.35 ± 0.30	75.13 ± 3.53	73.09 ± 5.92	78.25 ± 7.43
	CNN	97.66 ± 0.40	65.47 ± 3.84	65.44 ± 10.15	67.78 ± 8.39
	Transformer	97.62 ± 0.38	62.22 ± 4.34	57.50 ± 6.85	69.90 ± 10.65
	XGBoost	98.28 ± 0.26	77.22 ± 3.46	**85.74 ± 4.16**	70.29 ± 3.38
	SVM	97.19 ± 0.54	61.13 ± 2.63	64.71 ± 9.53	60.25 ± 8.83
	MLP	98.49 ± 0.34	77.03 ± 4.26	74.12 ± 3.10	80.58 ± 7.74
UCI SECOM	Ours (SAE + CSWE)	85.54 ± 7.36	**33.17 ± 6.29**	**49.05 ± 17.82**	**30.87 ± 16.01**
	XGBoost	87.55 ± 5.11	32.94 ± 3.36	45.24 ± 16.25	28.88 ± 6.66
	LightGBM	86.53 ± 7.36	30.86 ± 5.85	40.95 ± 11.11	28.05 ± 10.87
	Bagging	83.73 ± 9.23	23.51 ± 5.84	36.19 ± 18.34	20.33 ± 8.13
	Random Forest	87.45 ± 4.58	18.90 ± 11.83	27.62 ± 19.73	17.69 ± 10.94
	SVM	**91.66 ± 1.01**	9.67 ± 6.31	6.67 ± 4.36	18.82 ± 12.52
	MLP	91.24 ± 1.18	10.19 ± 5.59	7.62 ± 4.36	17.46 ± 9.44
	CNN	85.25 ± 9.73	14.08 ± 5.73	20.95 ± 17.46	15.86 ± 8.28
	Transformer	89.39 ± 1.47	14.78 ± 5.25	13.81 ± 5.41	16.67 ± 6.34

**Table 8:** Regression Performance on AI4I 2020 and NASA FD002 Datasets (Mean ± Std). The best results are highlighted in **bold**, and the second-best are underlined.

Dataset	Model	MSE	R^2^	MAPE (%)
AI4I 2020	Ours (SAE + ARSR)	**2584.88 ± 62.40**	**0.9196 ± 0.0047**	2.67 ± 0.02
	XGBoost	2735.70 ± 68.53	0.9149 ± 0.0043	2.72 ± 0.03
	LightGBM	2737.82 ± 68.79	0.9149 ± 0.0042	2.73 ± 0.03
	Bagging	2887.81 ± 92.82	0.9101 ± 0.0059	2.82 ± 0.04
	Random Forest	2887.92 ± 94.86	0.9101 ± 0.0060	2.82 ± 0.05
	SVR (Downsampled)	3651.86 ± 234.76	0.8866 ± 0.0070	2.81 ± 0.03
	MLP (Vanilla NN)	4191.91 ± 264.21	0.8696 ± 0.0105	3.22 ± 0.08
	CNN (1D) Fixed	2589.83 ± 65.09	0.9194 ± 0.0048	**2.66 ± 0.03**
	Tabular Transformer	2648.32 ± 80.67	0.9176 ± 0.0045	**2.66 ± 0.02**
NASA C-MAPSS FD002	Ours (SAE + ARSR)	**424.49 ± 36.03**	**0.7561 ± 0.0207**	32.14 ± 1.03
	XGBoost	434.01 ± 32.28	0.7507 ± 0.0185	**31.70 ± 1.01**
	LightGBM	435.13 ± 32.30	0.7500 ± 0.0185	32.01 ± 1.02
	Bagging	433.81 ± 34.72	0.7508 ± 0.0199	31.82 ± 1.16
	Random Forest	434.51 ± 32.58	0.7504 ± 0.0186	32.01 ± 1.15
	SVR	512.35 ± 32.87	0.7057 ± 0.0190	44.45 ± 2.99
	MLP	692.97 ± 82.49	0.6020 ± 0.0464	79.13 ± 11.86
	CNN (1D)	484.69 ± 41.63	0.7216 ± 0.0239	41.31 ± 3.48
	Tabular Transformer	970.55 ± 131.33	0.4425 ± 0.0754	114.26 ± 23.29

**Table 9:** Ablation Study Results for Classification (UCI SECOM) and Regression (NASA FD002). The best results are highlighted in **bold**, and the second-best are underlined.

Classification Task (UCI SECOM)				
**Variant**	**Acc. (%)**	**F1 (%)**	**Rec. (%)**	**Pre. (%)**
Ours (Full Model)	85.54 ± 7.36	**33.17 ± 6.29**	**49.05 ± 17.82**	**30.87 ± 16.01**
w/o Sparsity (AE)	82.93 ± 9.61	23.94 ± 2.75	40.00 ± 21.63	21.34 ± 6.03
w/o SAE (Raw)	**88.60 ± 3.36**	32.38 ± 4.48	40.48 ± 9.82	28.56 ± 5.23
w/o Partit. (Global)	86.02 ± 3.91	29.23 ± 6.13	43.33 ± 14.67	23.37 ± 6.09
**Regression Task (NASA FD002)**				
**Variant**	**MSE**	**R^2^**	**MAPE (%)**	
Ours (Full Model)	**424.49 ± 36.03**	**0.7561 ± 0.0207**	**32.14 ± 1.03**	
w/o Sparsity (AE)	485.99 ± 39.20	0.7208 ± 0.0223	35.72 ± 1.43	
w/o SAE (Raw)	452.24 ± 35.74	0.7402 ± 0.0205	35.11 ± 1.80	
w/o Partit. (Global)	474.45 ± 38.80	0.7275 ± 0.0221	37.17 ± 2.41	

**Table 10:** Performance Comparison on Industrial Data: Bagging vs. Region-Weighted Ensemble.The best results are highlighted in **bold**.

Metric	Bagging Ensemble	Ours (CSWE)
Precision	80.00%	**81.63%**
Recall	69.20%	**76.92%**
F1 Score	74.21%	**79.21%**

**Table 11:** MAPE of speed mean prediction and speed STD prediction. The best results are highlighted in **bold**, and the second-best are underlined.

	Algorithms	SUP	HSP	IP
MAPE of mean speed	LR	**5.43%**	0.89%	1.06%
	SVR	5.68%	**0.82%**	**0.51%**
	GPR	5.79%	1.63%	1.04%
	Regression tree	6.10%	1.15%	1.01%
	Bagging regression	5.48%	0.95%	0.89%
MAPE of speed STD	LR	57.12%	26.97%	97.66%
	SVR	**46.82%**	27.30%	190.10%
	GPR	100%	**23.54%**	99.77%
	Regression tree	66.27%	40.03%	112.43%
	Bagging regression	46.91%	26.04%	**46.45%**

## Data Availability

The data that support the findings of this study are available from the corresponding author, Li Liu, upon reasonable request.
